# Evaluation of Aminoglycoside and Non-Aminoglycoside Compounds for Stop-Codon Readthrough Therapy in Four Lysosomal Storage Diseases

**DOI:** 10.1371/journal.pone.0135873

**Published:** 2015-08-19

**Authors:** Marta Gómez-Grau, Elena Garrido, Mónica Cozar, Víctor Rodriguez-Sureda, Carmen Domínguez, Concepción Arenas, Richard A. Gatti, Bru Cormand, Daniel Grinberg, Lluïsa Vilageliu

**Affiliations:** 1 Departament de Genètica, Facultat de Biologia, Universitat de Barcelona, IBUB, CIBERER, Barcelona, Spain; 2 CIBBIM–Nanomedicina, Vall d'Hebron Institut de Recerca (VHIR), CIBERER, Hospital Universitari Vall d’Hebron, Barcelona, Spain; 3 Departament d’Estadística, Facultat de Biologia, Universitat de Barcelona, Barcelona, Spain; 4 David Geffen/UCLA School of Medicine, Departments of Pathology & Laboratory Medicine, and Human Genetics, Los Angeles, United States of America; University Hospital S. Maria della Misericordia, Udine, ITALY

## Abstract

Nonsense mutations are quite prevalent in inherited diseases. Readthrough drugs could provide a therapeutic option for any disease caused by this type of mutation. Geneticin (G418) and gentamicin were among the first to be described. Novel compounds have been generated, but only a few have shown improved results. PTC124 is the only compound to have reached clinical trials. Here we first investigated the readthrough effects of gentamicin on fibroblasts from one patient with Sanfilippo B, one with Sanfilippo C, and one with Maroteaux-Lamy. We found that ARSB activity (Maroteaux-Lamy case) resulted in an increase of 2–3 folds and that the amount of this enzyme within the lysosomes was also increased, after treatment. Since the other two cases (Sanfilippo B and Sanfilippo C) did not respond to gentamicin, the treatments were extended with the use of geneticin and five non-aminoglycoside (PTC124, RTC13, RTC14, BZ6 and BZ16) readthrough compounds (RTCs). No recovery was observed at the enzyme activity level. However, mRNA recovery was observed in both cases, nearly a two-fold increase for Sanfilippo B fibroblasts with G418 and around 1.5 fold increase for Sanfilippo C cells with RTC14 and PTC124. Afterwards, some of the products were assessed through *in vitro* analyses for seven mutations in genes responsible for those diseases and, also, for Niemann-Pick A/B. Using the coupled transcription/translation system (TNT), the best results were obtained for SMPD1 mutations with G418, reaching a 35% recovery at 0.25 μg/ml, for the p.W168X mutation. The use of COS cells transfected with mutant cDNAs gave positive results for most of the mutations with some of the drugs, although to a different extent. The higher enzyme activity recovery, of around two-fold increase, was found for gentamicin on the ARSB p.W146X mutation. Our results are promising and consistent with those of other groups. Further studies of novel compounds are necessary to find those with more consistent efficacy and fewer toxic effects.

## Introduction

Lysosomal storage disorders (LSDs) are a group of more than 50 genetic disorders caused by the lack of degradation of substrates within lysosomes. Most are caused by mutations in genes coding for lysosomal hydrolases. The main symptoms are bone and/or joint disease, mental retardation and/or developmental delay and visceromegalia. Lysosomal storage disorders are mainly inherited in an autosomal recessive manner, but in a few cases they are X-linked [1].

Mutations causing LSDs include missense and nonsense changes, splicing mutations, deletions, insertions, etc. Nonsense mutations can be corrected by drugs that produce the readthrough of the premature termination codon (PTC) (reviewed in Ref. [2–3]). In the present work we studied the correction of nonsense mutations in fibroblasts from patients with three LSDs: Sanfilippo syndrome types B and C, and Maroteaux-Lamy syndrome. Moreover, we also performed in vitro corrections for other mutations in the genes responsible for these diseases and, also, in the *SMPD1* gene that causes Niemann-Pick A/B disease.

Sanfilippo syndrome or mucopolysaccharidosis III (MPS III) has four subtypes (A: OMIM 252900, B: OMIM 252920, C: OMIM 252930 and D: OMIM 252940), due to mutations in four genes that result in the inability to degrade the glycosaminoglycan heparan sulfate [4]. Clinically, the four subtypes are similar, with severe central nervous system (CNS) degeneration, accompanied by mild somatic manifestations. Mucopolysaccharidosis type IIIB is characterized by deficiency in *N*-acetyl-α-glucosaminidase (Naglu, EC: 3.2.1.50) activity, which leads to lysosomal accumulation of the glucosaminoglycan (GAG) heparan sulfate (HS). The enzyme is encoded by the *NAGLU* gene (NCBI RefSeq NM_000263.4), which maps to chromosome 17 and has six exons. Mucopolysaccharidosis type IIIC is due to mutations in the *HGSNAT* gene (NCBI RefSeq NM_152419.2), which encodes acetyl-CoA:α-glucosaminide N-acetyltransferase (EC 2.3.1.78). The gene, located on chromosome 8p11.1, contains 18 exons [5,6]. The enzyme catalyzes acetylation of the terminal glucosamine residues of heparan sulfate prior to its hydrolysis by α-N-acetyl glucosaminidase [7].

Maroteaux–Lamy syndrome, or mucopolysaccharidosis (MPS) VI (OMIM 253200), is caused by impaired activity of the lysosomal enzyme N-acetylgalactosamine-4-sulfatase (4-sulfatase, arylsulfatase B or ARSB, EC 3.1.6.1) [4], resulting from mutations in the *ARSB* gene (NCBI RefSeq NM_00046.3). The enzyme deficiency leads to the accumulation of harmful amounts of undegraded dermatan sulfate. Symptoms include short stature, hepatosplenomegaly, dysostosis multiplex, joint stiffness, corneal clouding, cardiac abnormalities and coarse facies, without intellectual impairment.

Niemann-Pick disease (NPD) type A/B is an autosomal recessive sphingolipidosis caused by lysosomal acid sphingomyelinase (ASM, E.C. 3.1.4.12) deficiency. Type A (OMIM 257200) is a fatal infantile neurovisceral form and type B (OMIM 607616) presents a purely visceral form and survival till adulthood [8]. The acid sphingomyelinase gene (*SMPD1*; NCBI RefSeq NM_000543.4) is composed of six exons and is located on chromosome 11p15.1–11p15.4 [8].

The use of drugs that produce a readthrough of premature stop codons could be used for nonsense mutations mainly in diseases with neurological symptoms for which enzyme replacement therapy is not an option. An advantage of this strategy is that, if successful, it can be applied to any disease, provided that the molecular cause is a primary nonsense mutation (i.e., in which the PTC results directly from a point mutation in the DNA) [9]. In the case of neurological lysosomal storage diseases, additional advantages are the potential penetrance through the blood brain barrier, the fact that a small increase in enzyme activity is sufficient to correct the phenotype and that the approach implies an oral, non-invasive, therapy. The most extensively studied approach involves readthrough by drugs affecting the ribosomal decoding site.

In recent years, several research groups have tried to use aminoglycoside antibiotics to suppress stop codons in cells from patients bearing nonsense mutations. Positive results of treatment with gentamicin in cell culture experiments were first reported for cystic fibrosis [10]. The efficacy of this approach in vivo was first demonstrated in mdx mice using subcutaneous injection of gentamicin [11]. Since then, different aminoglycoside antibiotics have been shown to suppress premature translation termination at nonsense codons, with efficacies varying from 1% to 25% in human cell lines and animal models [12,13]. In the case of lysosomal disorders, this treatment has been assayed in human fibroblasts and animal models for some diseases [2,14–19].

Although gentamicin and geneticin have been extendedly used for this purpose, other aminoglycoside and non-aminoglycoside compounds such as amikacin [20], kanamycin B analogues [21], zidovudine, adefovir, cisplatin [22], RTC13 and 14 [23] and derivatives [24], NB54 [25] and NB84 [17] have also been tested.

High-throughput screens identified PTC124 (a.k.a. as Ataluren (Translarna), trade name of PTC Therapeutics) [26], which is a small organic molecule with no antibiotic properties that can promote readthrough of disease-causing PTCs and does not affect termination at stop codons located at the end of coding sequences. Unlike aminoglycosides, PTC124 has no serious adverse side effects. Therefore, it has great potential for treating genetic diseases. PTC124 probably functions at a different location on the ribosome than aminoglycosides, because it is part of a distinct structural class of drugs. This compound has been used in several assays for different diseases with positive effects [27–30], although negative results have also been reported [31–33]. Clinical trials of this drug are underway for patients with cystic fibrosis (phase III), Duchenne muscular dystrophy (DMD) (phase II), and other diseases [34]. A phase III study for DMD and other diseases started in 2014 (NCT02090959). Very recently, Gregory-Evans et al. [35] showed the reversion of aniridia in a mouse model of the disease through treatment with a topical application of the drug formulation named START, which contains 1% powdered Ataluren.

Readthrough efficiency inversely correlates with translation-termination efficiency, and may be influenced by different factors including the identity of the PTC and its context. It has been reported that the three stop codons are not equally susceptible to readthrough and this can vary from one species to another. In eukaryotes, the UGA stop codon is more amenable to readthrough than UAG, which is followed by UAA [36,37]. In addition, the sequence context in which the stop codon lies plays an important role in readthrough efficiency, particularly the nucleotide in position +4 [38]. However, the impact of the +4 nucleotide is also affected by its surrounding context [38].

One of the mechanisms that specifically regulates the level of PTC-bearing transcripts is nonsense-mediated decay (NMD), a quality-control process that detects and degrades such transcripts to prevent the synthesis of unstable proteins that might be deleterious for the cell. PTC readthrough compounds may increase the stability of mutant RNA by limiting NMD. In fact, several papers have reported that gentamicin and other readthrough agents inhibit NMD and thereby increase the amount of PTC-containing RNAs [39,40].

In this study, we first investigated the readthrough effects of gentamicin on fibroblasts from one patient with Sanfilippo B, one with Sanfilippo C, and one with Maroteaux-Lamy. Geneticin and five non-aminoglycoside (PTC124, RTC13, RTC14, BZ6 and BZ16) readthrough compounds (RTCs) were also assayed for the two cases that did not respond to gentamicin. Additionally, different products were tested on seven mutations for which fibroblasts were not available using the coupled transcription/translation system (TNT) and COS cells transfected with cDNA bearing nonsense mutations. We obtained positive results for some drugs and mutations, encouraging us to continue with research on this therapeutic strategy.

## Material and Methods

### Mutations and patients’ fibroblasts

The primary nonsense mutations analysed in this study are listed in Table 1. They all were identified in previous studies [6,41–49]. Several of them were found in Spanish patients.

**Table 1:** Studied nonsense mutations.

Gene	Disease	Mutation		Exon^1^	Stop codon^2^	References
		c.DNA	Protein			
**FIBROBLASTS**						
*ARSB*	Maroteaux-Lamy	c.966G>A	p.W322X	5 (8)	(G) UGA (G)	[41]
*NAGLU*	Sanfilippo B	c.503G>A	p.W168X	2 (6)	(C) UAG (A)	[42]
		c.1696C>T	p.Q566X	6 (6)	(G) UAG (G)	[43]
*HGSNAT*	Sanfilippo C	c.1150C>T	p.R384X	12 (18)	(U) UGA (G)	[6]
**cDNAs**						
*ARSB*	Maroteaux-Lamy	c.438G>A	p.W146X	2 (8)	(A) UGA (C)	[44]
		c.1507C>T	p.Q503X	8 (8)	(A) UAG (U)	[45]
*SMPD1*	Niemann-Pick A/B	c.503G>A	p.W168X	2 (6)	(C) UAG (G)	[46]
		c.939C>A	p.Y313X	2 (6)	(G) UAA (C)	[46]
		c.1321C>T	p.R441X	4 (6)	(C) UGA (A)	[47]
*HGSNAT*	Sanfilippo C	c. 607C>T	p.R203X	6 (18)	(U) UGA (G)	[48]
		c.1209G>A	p.W403X	12 (18)	(G) UGA (C)	[49]

^1^Exon bearing the nonsense mutation (total number of exons of the corresponding gene)

^2^In brackets, note the nucleotides immediately 5’ or 3’ to the stop codon. Point mutation resulting in PTC is underlined.

Fibroblasts from a Spanish MPSVI patient (patient ML4, genotype p.W322X/c.427delG) [41], from a Spanish SFB patient (patient SFB1, genotype p.W168X/p.Q566X, named P5 in Matalonga et al. [43]), and from a French SFC patient (patient SFC13, genotype p.R384X/c.1542+4dupA) [50], were available and were used in some of the experiments.

The three *SMPD1* mutations were found in Spanish Niemann-Pick patients [46]. The other four mutations were obtained from the literature.

### Ethics statement

The Bioethics Committee of the Universitat de Barcelona released a favourable bioethical statement regarding the present research. Patients were encoded to protect their confidentiality and written informed consent was obtained.

### Culture and treatment of patients’ fibroblasts

Fibroblasts were cultured in DMEM supplemented with 10% FBS and 1% penicillin/streptomycin (Gibco) at 37°C and 5% CO_2_, in six-well plates, except for the NAGLU activity measurements, for which 100 mm plates were used.

For the readthrough experiments, gentamicin (300–700 μg/ml), G418 (75 μg/ml), PTC124 (20 μM), RTC13, RTC14, BZ6 and BZ16 (30 μM) were added to the medium without antibiotics. Fresh media and drugs were replaced every 24 h and cells were harvested after 3–4 days of treatment, at which point mRNA was quantified and enzyme activities were analysed. Three replicates were assayed for each condition.

For the indirect immunofluorescence studies of 4-sulfatase, WT fibroblasts and fibroblasts from the MPS VI patient ML4 [41] were grown on coverslips in 12-well plates and fixed after 6 days of treatment with increasing concentrations of gentamicin (500, 1000 and 1500 μg/ml). Anti-human ARSB and Lamp-2 (a marker of lysosomes and late endosomes) antibodies were used, as in Garrido et al. [51]. Briefly, the primary antibodies used to label the lysosomes were a sheep antibody against 4-sulfatase and the mouse anti-human Lamp-2 monoclonal antibody H4B4 (Developmental Studies Hybridoma Bank, University of Iowa, Department of Biological Sciences, Iowa City, IA, USA). The secondary antibodies were a donkey FITC anti-sheep IgG (Jackson Immunoresearch Laboratories, Inc., West Prove, PA, USA) and goat AlexaFluor 660 antimouse IgG from Molecular Probes (Invitrogen). Images were captured with an Olympus Fluoview FV300 confocal microscope and analysed with Fluoview FV500 image software. Experiments were repeated at least three times to ensure reproducibility. Autofluorescence and secondary antibody control tests were performed.

### Enzyme assays

Protein concentration was determined by the Lowry method. The activity measurements were performed in duplicate (at least) in all cases. The activity of another lysosomal enzyme (β-hexosaminidase) was measured as a control (data not shown).

Acid sphingomyelinase and acetyl-CoA:α-glucosaminide N-acetyltransferase activities were analysed using the fluorogenic substrates 6-hexadecanoylamino-4-methylumbelliferyl-phosphorylcholine and 4-methylumbelliferyl-β-d-glucosaminide (Moscerdam Substrates, Oegstgeest, The Netherlands), respectively. Measurements were performed in a Modulus Microplate Multimode Reader (Turner Biosystems, Sunnyvale, CA, USA), following the manufacturer's instructions. The 4-sulfatase activity was determined by spectrophotometric quantification of p-nitrocatecol (absorbance at 515 nm) produced by hydrolysis of the substrate p-nitrocatecol sulfate dipotassium salt (Sigma-Aldrich, St. Louis, USA).

For the α-N-acetylglucosaminidase assay, 20 μl of 200 mM sodium acetate buffer, pH 4.3, was combined with 40 μl of the fluorogenic substrate 4-methylumbelliferyl-2-acetamido-2-deoxy-α-D-glucopyranoside (Calbiochem, Merck, Darmstadt, Germany) 2 mM, and 20 μl of cell lysate was added. The mixture was incubated for 2 hours at 37°C and the reaction was stopped with 1 ml of glycine buffer 100 mM pH 10.4. Measurements were performed in a fluorometer (excitation 365 nm, emission 450 nm).

### mRNA quantification

The levels of *HGSNAT* and *NAGLU* transcripts in patients' fibroblasts were analysed by qRT-PCR in a LightCycler 480 instrument (Roche Applied Sciences, Indianapolis, IN, USA). For RNA isolation, cultured patients' fibroblasts were harvested and RNA was obtained using a High Pure RNA Isolation Kit (Roche Applied Sciences), following the manufacturer's recommendations. Concentrations were determined using the NanoDrop ND-1000 spectrophotometer (NanoDrop Technologies, Wilmington, DE, USA). RNA samples were stored at −80°C until analysis. The RNA obtained was retrotranscribed using a High-Capacity cDNA Archive Kit (Applied Biosystems, Foster City, CA) and real time-PCR was performed using the LightCycler 480 II system and the Universal Probe Library (Roche Applied Science). Gene assays were designed using Universal ProbeLibrary Assay Design Center software (Roche Applied Science). Sequence of primers and probes used are available upon request. Human-specific Taqman Gene Expression assays for *SDHA (succinate dehydrogenase complex*, *Hs00417200_m1)* and *HPRT1 (hypoxantine phosphoribosyltransferase 1*, *Hs99999909_m1)* (the latter not shown) genes were used as endogenous controls to normalise the relative amounts of mRNA. These genes were selected because they were stably expressed under the experimental conditions. The Roche LightCycler 480 software was used to performed advanced relative quantification analysis of gene expression, according to the LightCycler 480 instrument operator’s manual.

### Nonsense-mediated RNA decay

For nonsense-mediated mRNA decay experiments, fibroblasts from SFB and SFC patients were seeded on six-well plates and cultured in the absence or presence of 1 mg/ml of cycloheximide (CHX) for 6 h. Cycloheximide, an inhibitor of protein synthesis, is one of the typical compounds used to assay for the occurrence of NMD. Total RNA was isolated and retrotranscribed as described above. A specific PCR amplification was performed to obtain fragments including mutations p.W168X (*NAGLU*) or p.R384X (*HGSNAT*), which were digested by restriction enzymes *Bmp*I and *Xho*I, respectively, and the fragments were then separated by electrophoresis. Quantification of the relative intensity of the electrophoretic bands was performed using IMAGE LAB5.1 Analysis Software (Bio-Rad, Hercules, CA, USA). Amplification of the *GAPDH* cDNA was used as a control.

### Site-directed mutagenesis

The nonsense mutations were introduced in the wild type full-length cDNA of the corresponding gene cloned in the pcDNA3.1 expression vector by PCR-based site-directed mutagenesis using the QuikChange II XL Site-Directed Mutagenesis Kit (Stratagene Cloning Systems, La Jolla, CA, USA), according to the manufacturer’s instructions. All constructs were resequenced to ensure that no spurious mutations had been introduced.

### Readthrough drugs and coupled transcription/translation assay

The TNT Quick Coupled Transcription/Translation System (Promega, Madison, WI, USA) was used for *in vitro* protein synthesis, as described by Sánchez-Alcudia et al. [32]. To assay the effect of the readthrough drugs, a range of different concentrations of G418 (0.25–10 μg/ml; Gibco, Carlsbad, CA, USA), gentamicin (2.5–20 μg/ml; Gibco), PTC124 (1.5–10 μM; Excenen Pharmatech Co., Ltd, Guangzhou City, China), RTC13 (10–40 μM) and RTC14 (10–40 μM) [23], BZ6 (10–40 μM)] and BZ16 (10–40 μM) [24,52,53] were added to the coupled TNT reaction. Mutation *SMPD1* (p.Y313X) was used to choose the best concentrations for each product. Readthrough efficiency was calculated as the amount of full-length protein produced relative to the sum of the truncated protein plus the full-length protein, expressed as percentages.

### COS7 cell culture and transfection

COS7 cells were a gift from the URFOA group at the Institut Hospital del Mar de Investigacions Mèdiques (IMIM), which were originally obtained from ATCC (CRL-1651). They were cultured in DMEM supplemented with 10% FBS and 1% penicillin/streptomycin (Gibco) at 37°C and 5% CO_2_. For transfection with the cDNAs, COS7 cells were seeded on six-well plates. When the cells were at 80–90% confluence, 1 μg of the plasmid bearing either the wild type or the mutant cDNA (corresponding to the genes *ARSB*, *SMPD1* or *HGSNAT*) was introduced using lipofectamine 2000 (Invitrogen, Life Technologies, Paisley, UK), following the manufacturer’s instructions. As a negative control, the pcDNA3.1 empty vector was transfected. Different concentrations of G418 (50–100 μg/ml), gentamicin (500–1000 μg/ml) and PTC124 (20–60 μM) were added 4 h post-transfection. Fresh media and drugs were replaced every 24 h. Cells were harvested 48 h after treatment for *ARSB* and *SMPD1* and 72 h after treatment for *HGSNAT*, and centrifuged. The pellets were washed twice with PBS and stored at -80°C until enzyme activity analyses were performed. Two independent transfection experiments were performed for each mutation (together with the wild type construct and the negative control). The residual enzyme activity of the mutant alleles was analysed as described above. Results were expressed as a percentage of the mean activity of the wild-type construct transfected in the same experiment. The activity of the negative control was subtracted.

### Statistical analyses

Statistical analyses were performed using the Student’s t-test. It should be noted that this test was recently validated for extremely small sample sizes [54]. A p-value < 0.05 was predetermined as significant.

## Results

### Readthrough treatment of patients’ fibroblasts

**Fig 1:**
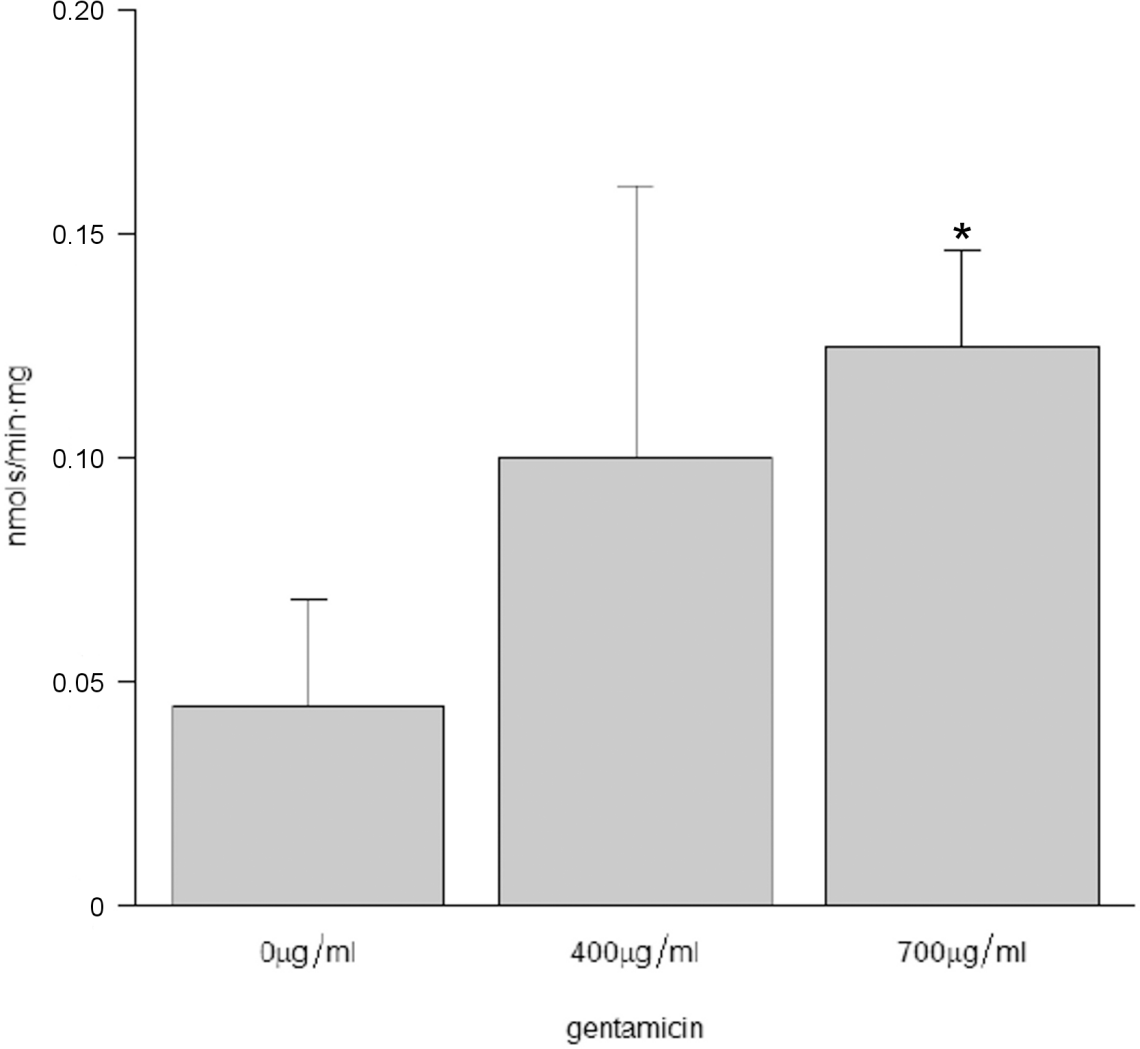
Effect of gentamicin treatment on ARSB activity in fibroblasts. Fibroblasts from a MPSVI patient with genotype W322X/c.427delG were treated for 96 hours at the indicated concentration. Each experiment is an average of three replicates. * p < 0.05, compared to 0 μg/ml (untreated).

**Fig 2:**
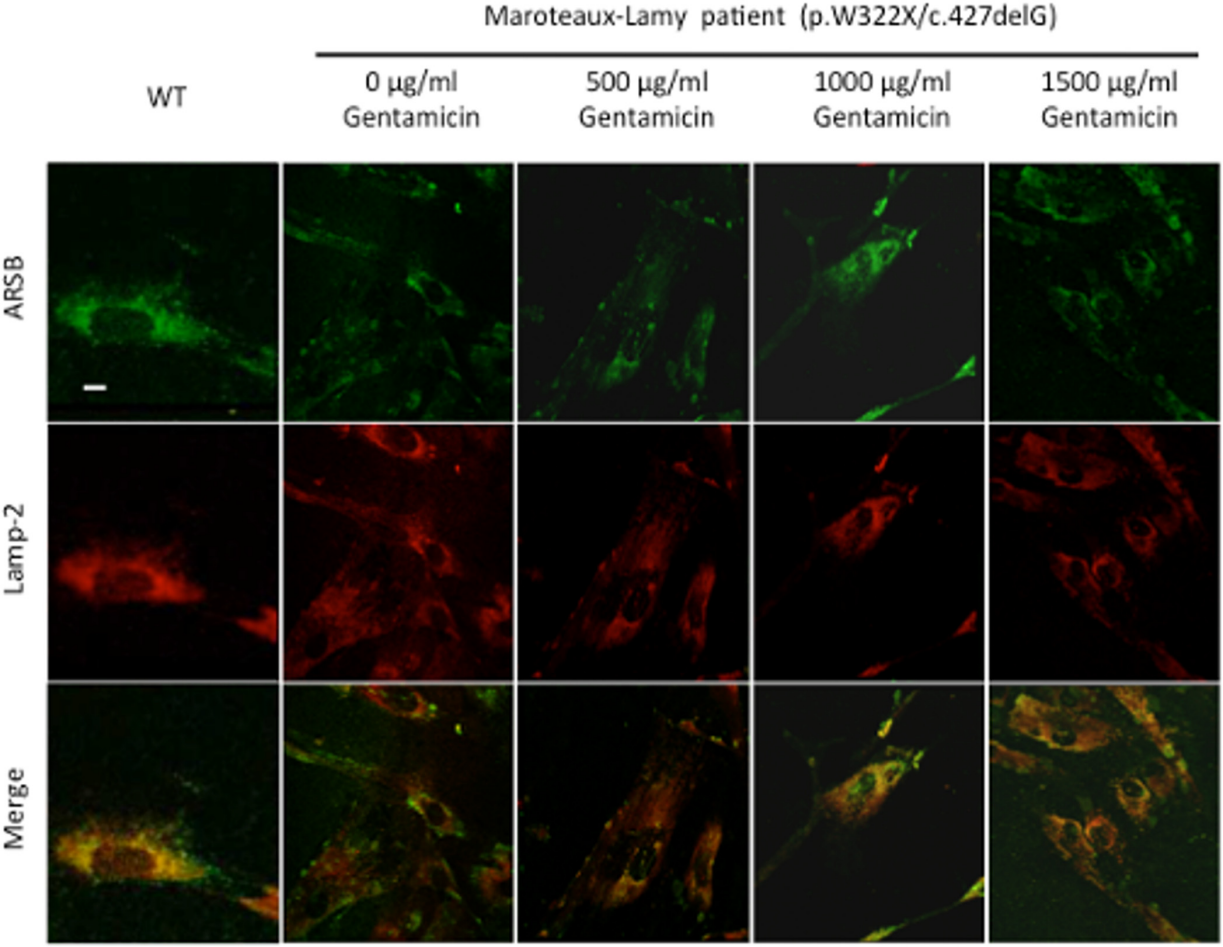
Confocal microscopy analysis of fibroblasts treated with gentamicin. Subcellular localization of the ARSB protein by indirect immunofluorescence in WT fibroblasts and in those from the MPSVI patient with genotype W322X/c.427delG. Fibroblasts were treated with the indicated gentamicin concentrations. ARSB protein was detected using a FITC-conjugated secondary antibody (green). Co-localization with Lamp-2 (an endogenous marker of lysosomes and late endosomes, marked in red) is shown in the overlay. The three replicates gave similar results. Magnification: 600X. The scale bar represents 20 μm.

After 96 hours of treatment with gentamicin, ARSB activity in patient ML4 fibroblasts (bearing the p.W322X mutation) was twice (226%) to three times (282%) that of untreated fibroblasts in the 400 and 700 μg/ml conditions, respectively (Fig 1). When we compared treated to untreated cells, significant differences were only found between cells treated with gentamicin 700μg/ml and untreated cells (p = 0.031). The enzyme activity achieved corresponds to 1–4% of that found in wild-type fibroblasts [41]. Also, fibroblasts of this patient were studied to detect the localisation of the mutant ARSB protein by immunofluorescence labelling. As shown in Fig 2, the amount of protein and the co-localisation with the lysosomal marker (LAMP2) was diminished in the patient compared with the WT fibroblasts. Both parameters were partially recovered by exposure to 1000 and 1500 μg/ml of gentamicin.

Fibroblasts from the Sanfilippo B and C patients showed no increase in enzyme activity upon gentamicin treatment. Thus six other readthrough compounds (G418, PTC124, RTC13, RTC14, BZ6 and BZ16) were assayed at different concentrations, but none of them led to positive recovery of enzyme activity.

**Fig 3:**
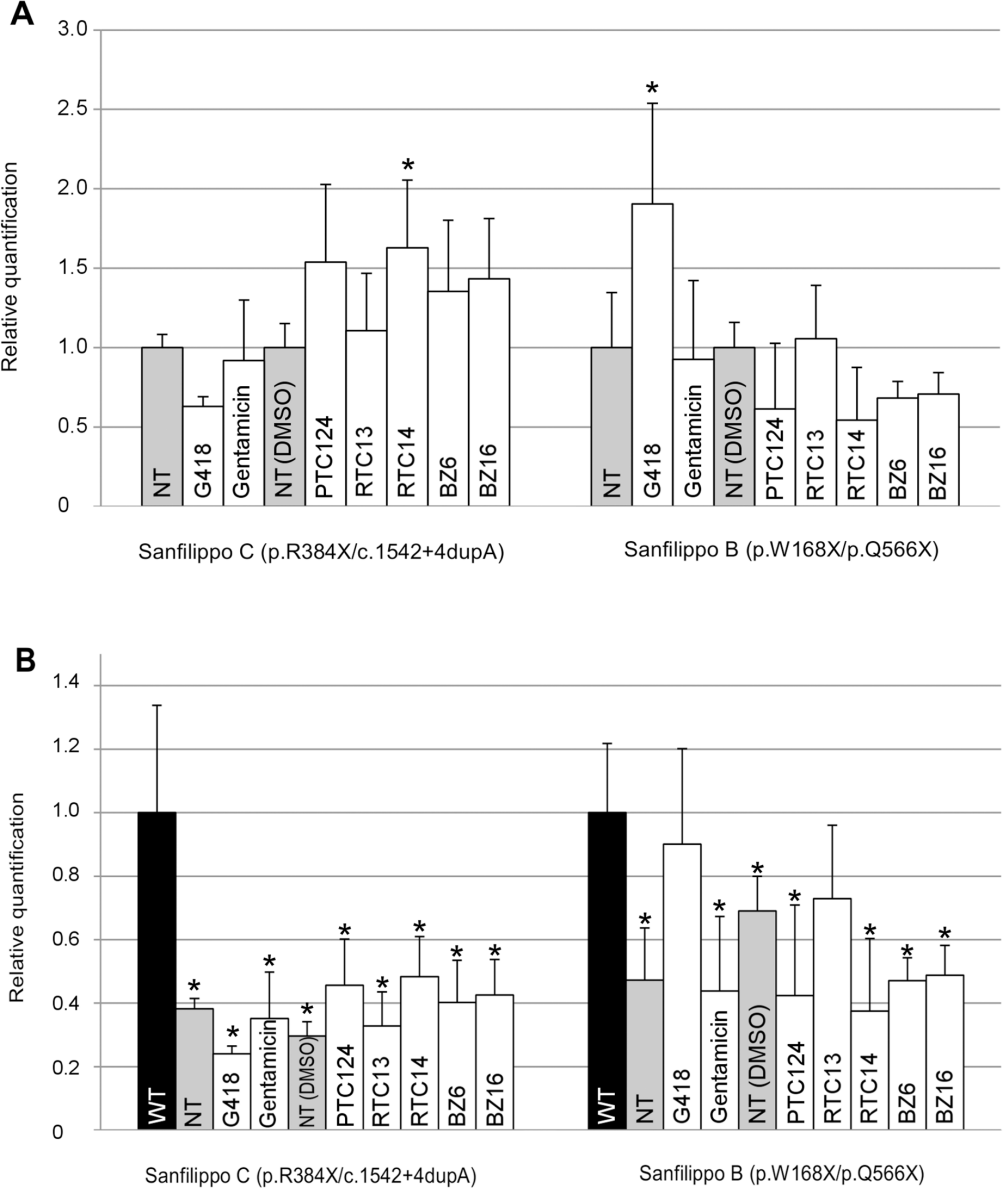
Quantification by qRT-PCR of *HGSNAT* (Sanfilippo C) and *NAGLU* (Sanfilippo B) mRNA levels in untreated and treated cultured fibroblasts. The concentrations of the products were G418 (75 μg/ml), gentamicin (300 μg/ml), PTC124 (20 μM), RTC13, RTC14, BZ6 and BZ16 (30 μM). (**A**) The Y-axis represents the results of the relative quantification of mRNA levels, normalized with respect to the untreated fibroblasts (NT), considered as 1. For PTC124, RTC13, RTC14, BZ6 and BZ16 the untreated samples included dimethyl sulfoxide (DMSO), since these products were dissolved in DMSO. (**B**) The Y-axis represents the results of the relative quantification of mRNA levels, normalized with respect to the wild-type fibroblasts (black bars). Data represent the mean ± SD of three experiments. Syndromes and genotypes of the different fibroblasts are indicated. * p < 0.05, compared to untreated cells (A) or to WT (B). (Note that: only increases in mRNA level were considered and that in part B, the relevant results are those that reached RNA levels similar to those of WT, i.e., that are not significantly different from those of WT.)

In fibroblasts from these two patients, mRNA levels (of the *NAGLU* and *HGSNAT* gene, respectively) were quantified after treatment with the seven compounds mentioned above (Fig 3). Note that in these experiments the concentration of gentamicin was lower than that used for the experiments shown in Figs 1 and 2, to facilitate comparisons with results by other authors (for example, reference [32]). Treatment of Sanfilippo B fibroblasts with G418 yielded an almost two-fold increase in mRNA levels (p = 0.025; Fig 3A). For Sanfilippo C cells, RTC14 and PTC124 gave the best results (1.6 and 1.5-fold increase, respectively, although only the former reached significance, p = 0.037) (Fig 3A). When compared with WT, they reached 45–50% (Sanfilippo C, treated with PTC124 or RTC14) to 75–90% (Sanfilippo B, treated with RTC13 or G418, respectively) of WT levels (Fig 3B). The treatment of Sanfilippo B cells with RTC13 or G418 gave results that did not significantly differ from those of WT (p = 0.086 and p = 0.330, respectively). It would have been interesting to assess the level of translation of these mRNAs, but the lack of appropriate antibodies and the technical problems we had with those that were available precluded obtaining data on this issue.

**Fig 4:**
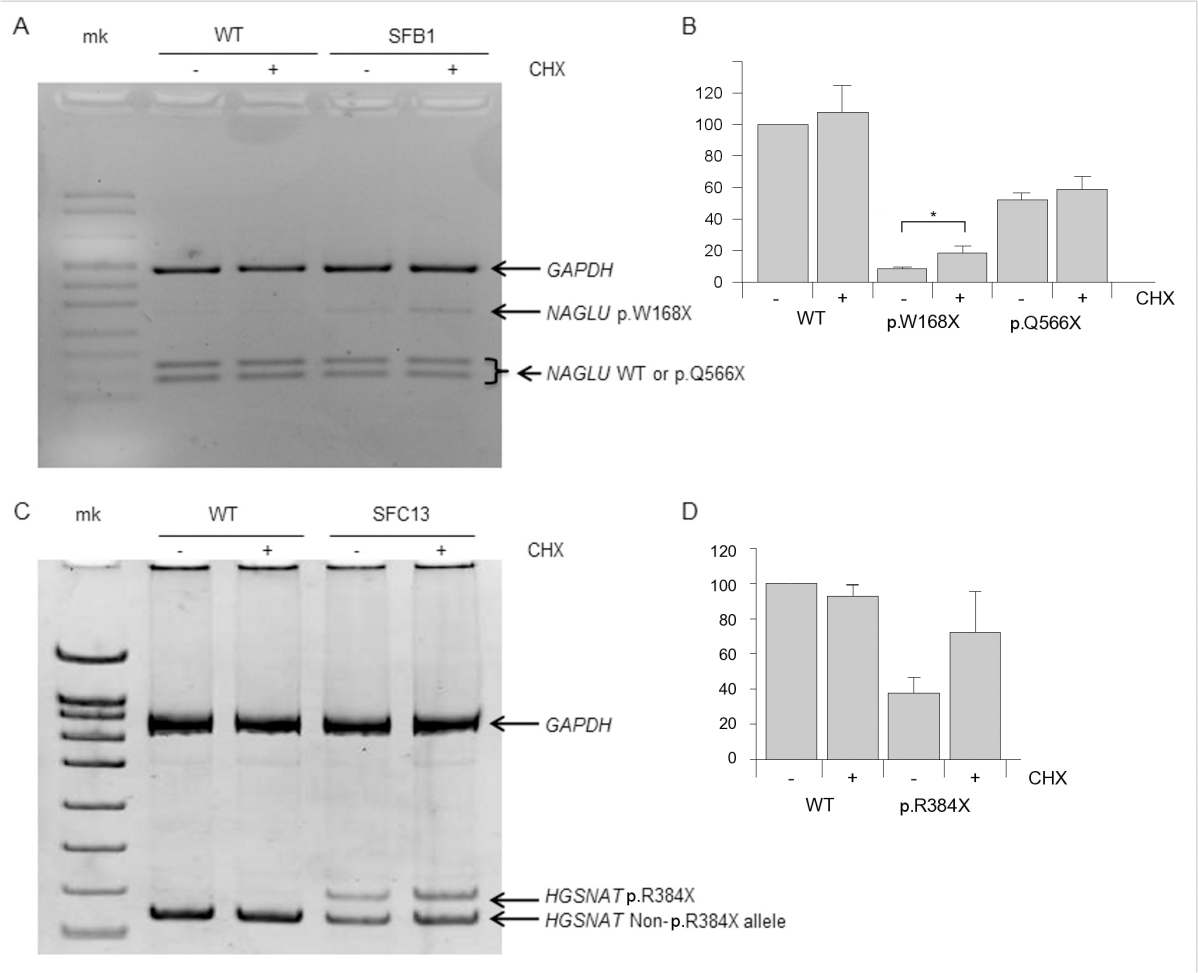
NMD analysis. (**A**) Agarose gel electrophoresis analysis of a cDNA fragment of the *NAGLU* gene (298-bp long), which includes position c.503 (corresponding to mutation p.W168*), amplified from WT fibroblasts or fibroblasts from patient SFB1 (genotype: p.W168*/p.Q566*), grown in the absence (-) or presence (+) of cycloheximide (CHX) and digested with *Bmp*I. Alleles not bearing the mutation (WT or p.Q566*) were digested, yielding two fragments of 162 and 136 bp. GAPDH was used as a control gene. mk: marker. The experiment was repeated three times, one of which is shown. (**B**) Quantification of the relative amounts of the bands shown in A and in two additional replicates. * = p < 0.05. (**C**) Agarose gel electrophoresis analysis of a cDNA fragment of the *HGSNAT* gene (140-bp long), which includes position c.1150 (corresponding to mutation p.R384*), amplified from WT fibroblasts or fibroblasts from patient SFC13 (genotype: p.R384*/c.1542+4dupA), grown in the absence (-) or presence (+) of cycloheximide (CHX) and digested with *Xho*I. Alleles not bearing the mutation (WT or c.1542+4dupA) were digested, yielding two fragments of 121 and 19 bp (not shown in the gel). GAPDH was used as a control gene. mk: marker. The experiment was repeated three times, one of which is shown. (**D**) Quantification of the relative amounts of the bands shown in C and in two additional replicates.

To test whether the stop codon mutations could cause a decrease in mRNA levels through the nonsense-mediated decay mechanism, fibroblasts were grown in the presence of cycloheximide, and RT-PCR was performed. As shown in Fig 4, a significant increase in the mRNA levels of the allele bearing the stop mutation *NAGLU* p.W168X (Fig 4A and 4B) was observed after CHX treatment. For the *HGSNAT* p.R384X allele, a clear increase was also observed, with borderline significance (p = 0.07) (Fig 4D). The allele bearing the *NAGLU* Q566X mutation (Fig 4A and 4B) did not show any significant increase, as expected, since it lies in the last exon of the gene.

### *In vitro* readthrough

**Fig 5:**
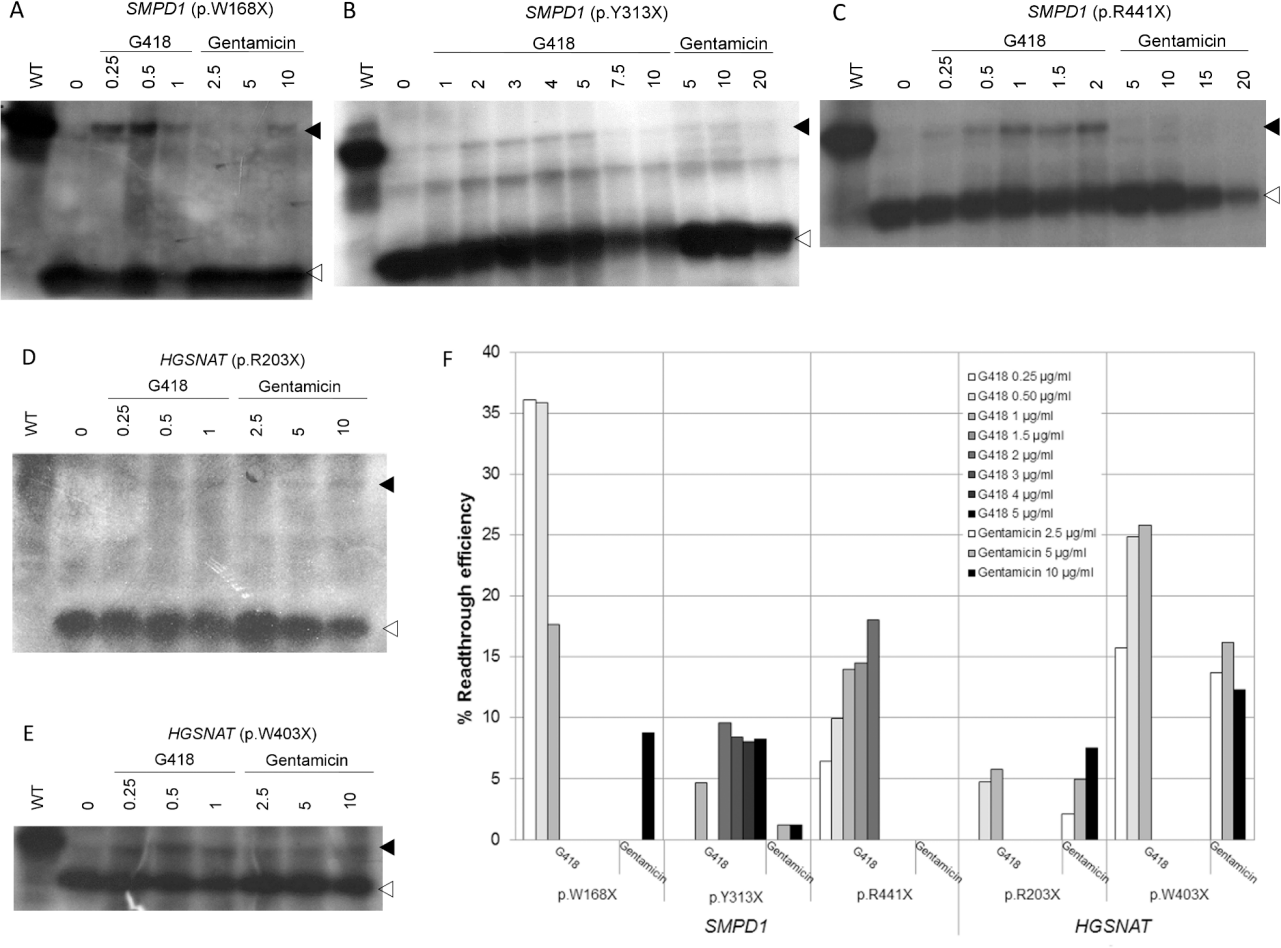
Effect of G418 and gentamicin on in vitro readthrough of nonsense mutations in the *SMPD1* and *HGSNAT* genes in the TNT system. The figure shows representative experiments for the indicated *SMPD1* (**A-C**) and *HGSNAT* (**D-E**) nonsense mutations. WT, wild-type construct. Black arrowhead, full-length protein. White arrowhead, truncated protein. Concentrations are in μg/ml. (**F**) Quantification of the full-length protein synthesized from each mutant construct. The percentage of full-length protein relative to the sum of full-length plus truncated proteins is shown for each aminoglycoside treatment and for the indicated concentration.

For mutations for which fibroblasts were not available, we tested the effect of the seven different products using a mammalian-coupled TNT assay of cDNAs bearing these nonsense mutations. In the absence of treatment, truncated proteins of the expected size for each of the mutations were synthesised. Recovery of the full-length protein was observed for some of the mutants with G418 (geneticin) or gentamicin (Fig 5), while no recovery was observed with PTC124, RTC13, RTC14, BZ6 and BZ16 (not shown). In particular, for the three *SMPD1* mutations, clear recovery was observed with G418 treatment at different concentrations, while gentamicin had a lower effect on mutations p.W168X and p.Y313X and no effect on p.R441X (Fig 5A–5C). The best results were around 35% recovery for the p.W168X mutation with 0.25 μg/ml of G418, and around 18% recovery for mutation p.R441X after treatment with 2 μg/ml G418 (Fig 5F). Additionally, positive results were obtained for the two *HGSNAT* mutations (p.R203X and p.W403X), the latter showing better results (Fig 5D and 5E), with recovery of around 25% with G418 at 0.5 and 1 μg/ml (Fig 5F). Positive results with gentamicin were also observed for these two *HGSNAT* mutations, with a maximum of 16% recovery for p.W403X with 5 μg/ml of gentamicin (Fig 5F). No recovery was found for the *ARSB* mutations (p.W146X and p.Q503X).

### Enzyme activity in transfected COS cells

**Fig 6:**
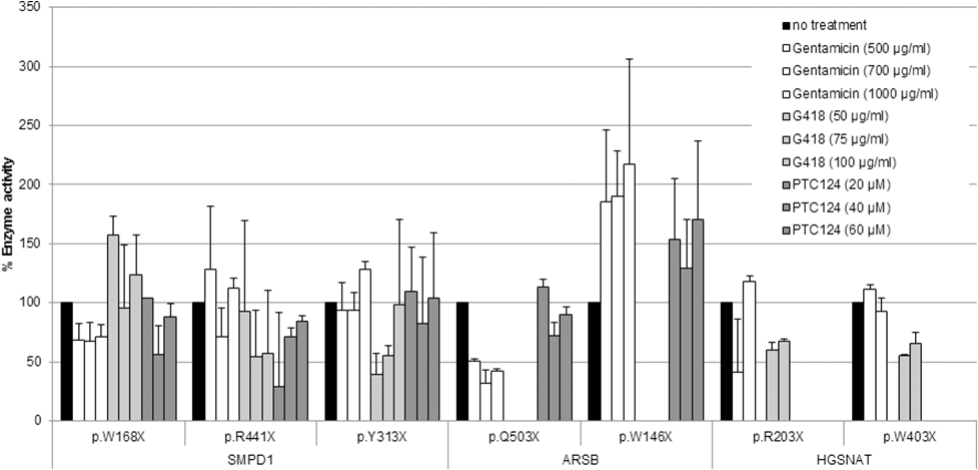
Effect of different readthrough compounds on the enzyme activity in COS cells transfected with cDNAs bearing the indicated nonsense mutation. The results are the mean of at least two experiments performed in duplicate. Pattern codes for compounds and concentrations are indicated in the inset.

For mutations for which fibroblasts were not available, the possible recovery of enzyme activity was analysed in COS cells transfected with the mutated cDNA. In particular, for *SMPD1* we analysed the three mutations found in Spanish patients [46]. For *ARSB* and *HGSNAT* we decided to include two mutations from the literature for each gene, predicted to generate either a short or a long truncated protein. For this assay, only gentamicin, geneticin and PTC124 were used. The best results were obtained for the *ARSB* p.W146X mutation with gentamicin, for which an almost two-fold increase was obtained (Fig 6). A positive result for this mutation was also found with PTC124 treatment, although to a lesser extent. A moderate increase in activity (around 20 to 50%, with borderline significance) was found for the following mutations and treatments: *SMPD1* p.W168X treated with G418; *SMPD1* p.Y313X treated with gentamicin; *ARSB* p.503X treated with PTC124; and *HGSNAT* p.R203X and p.W403X treated with gentamicin (Fig 6).

## Discussion

Correction of nonsense mutations with small molecules that interfere with ribosomal function and alter the translation of mRNA by bypassing PTCs could be an interesting therapeutic option for monogenic diseases caused by this type of DNA mutation. Such correction has been reported as being successful for several diseases and compounds and several studies have validated this strategy using animal models. However, a critical point to be taken into account is whether the efficacy of readthrough is clinically relevant. In this regard, lysosomal storage disorders could be a good option since it has been reported that small amounts of functional protein (5–10% of enzymatic activity) may be considered therapeutically relevant in several LSDs [4]. This same principle applies for ataxia-telangiectasia [9].

Herein we first assayed gentamicin in fibroblasts from three patients, each of them affected by a different lysosomal disease: Sanfilippo B, Sanfilippo C and Maroteaux-Lamy. Only the Maroteaux-Lamy fibroblasts responded to this treatment. For the other two fibroblasts the study was extended using six additional readthrough drugs, with negative results for all compounds. Additionally, mutations for which fibroblasts were not available were analysed both by an in vitro transcription/translation system (TNT) and by measuring the enzyme activity in COS transfected cells.

Gentamicin is the most frequent PTC-specific compound used to date. In our study, we found a significant positive result for enzyme activity in the Maroteaux-Lamy fibroblasts. However, due to the small sample sizes, this significance should be considered with caution. This positive result prompted us to perform indirect immunofluorescence studies on these cells, which showed improved trafficking of the mutant protein. For studies at the cDNA level, gentamicin produced some recovery of the full-length protein in the TNT assay and of the enzyme activity in transfected COS cells, for some of the mutations. However, it should be noted that the main recovery of activity of ARSB p.W146X did not correlate with the TNT result for this mutation. This could be due to technical problems encountered with this construct in the TNT assays.

G418 showed good results in the TNT experiments for different mutations. Interestingly, this compound also showed good recovery of the mRNA levels for Sanfilippo B fibroblasts bearing the p.W168X/p.Q566X genotype, although no increase in enzyme activity was observed, suggesting that the missense protein generated by the readthrough was not active. This increase in mRNA levels could be due to improved mutant RNA stability as a consequence of a reduction in NMD efficacy. We were able to demonstrate that the NMD mechanism was partially responsible for the reduction in RNA levels in the alleles bearing nonsense mutations located in the NMD-competent region of the gene. Thus, a possible strategy would be treatment with readthrough drugs, together with NMD inhibitors, as suggested by Bordeira-Carrico et al. [55]. However, the toxicity of the different compounds, including G418, argues against their therapeutic potential. Some authors also found good results for G418 [23,32,56,57].

PTC124 was identified as a readthrough drug over 10 years ago and was reported as non-toxic and orally bioavailable [26]. It appeared as the most promising compound for the correction of nonsense mutations, but there was also controversy about the results. Also, it does not cross the blood–brain barrier efficiently and is a poor candidate for adjunctive readthrough therapy with replacement proteins for LSDs. In the present study, we found some recovery of ARSB activity in transfected COS cells and of RNA levels in one of the Sanfilippo C fibroblasts. The effect of PTC124 on one of the *ARSB* mutations studied here, p.Q503X, was previously analysed in patients’ fibroblasts by Bartolomeo et al. [18], who found no recovery of activity. Whereas Peltz et al. [34], from PTC Therapeutics Inc., consider that there are multiple, independent demonstrations of Ataluren’s (PTC124) nonsense suppressing activity, other authors question this statement. McElroy et al. [33] failed to find evidence of activity for PTC124. The slight effects found in our study are consistent with those who found that this compound is, at least, not as effective as it was claimed when it was discovered. The result of the ongoing clinical trials will be very interesting.

We also assayed several compounds generated at UCLA: RTC13 and RTC14, BZ6 and BZ16 [23,24,52,53]. We found some recovery of RNA levels with RTC14 and BZ16 in Sanfilippo C fibroblasts (particularly with the former). Positive results for RTC13 and RTC14 have been found for numerous mutations in the ATM gene [23], DMD [23,58], and other genes such as collagen VII [9]. BZ16 (a derivative of RTC13) and RTC14 were also shown to increase *XPC* mRNA expression in skin fibroblasts from xeroderma pigmentosum (Group C) patients [24,53]. Our results are rather modest compared to those published data.

Response to this type of treatment is variable. In general, it is not a very robust response, a fact that was noted in early papers describing "nonsense suppression" in the 1960s. A possible explanation for the variability in the results is the different PTCs involved and the context surrounding them (basically, the fourth nucleotide). The UGA codon followed by a C has been reported to be the best combination for readthrough or efficiency as a STOP codon [37,38]. In the present study, only two out of the 11 mutations had this optimal combination (see Table 1). One of them, *HGSNAT* p.W403X, showed the best results in the TNT assay with gentamicin and G418, and the other one, *ARSB* p.W146X, showed the best recovery of activity in transfected COS cells after treatment with gentamicin and PTC124. Some authors suggest that besides the nucleotide in the 4^th^ position, the first nucleotide 5’ of the PTC is also important, with U being more susceptible to promote readthrough, independent of the stop codon itself [59]. In the present study, 2 out of the 11 mutations bear a U in this position (both in the *HGSNAT* gene: p.R203X and p.R384X). While they also carry the UGA stop codon (reported to be the best by several authors), none of them carry the C in the 4^th^ position, which should make them all sub-optimal, according to Floquet et al. [53].

For one of the mutations (*ARSB* p.W322X) we showed a significant increase in protein level after gentamicin treatment, through immunofluorescence studies in fibroblasts from a patient who bore a null mutation in the other allele. This experiment also showed that the protein was in the expected location within the cell, i.e. the lysosomes, through colocalisation analyses using the lysosomal marker Lamp-2. Few studies on readthrough drugs have used this type of technique to validate the correction [23,24]. In the case of lysosomal diseases, Bartolomeo et al. [18] analysed the reduction in lysosomal size in Maroteaux-Lamy fibrolasts after treatment with PTC124. However, the data were obtained by electron microscopy and no information on the protein was available. It should be noted that, despite the increased ARSB protein signal that was observed in the lysosome after gentamicin treatment, we did not detect a corresponding increase in enzyme activity. It could be speculated that the protein generated by readthrough is an inactive missense variant, albeit with improved folding that allows its trafficking to the lysosome.

As a summary of the relevant findings of this study, we found positive results for ARSB activity and enzyme localization in Maroteaux-Lamy fibroblasts treated with gentamicin. Additionally, an increase of mRNA levels was obtained with several products in Sanfilippo B and Sanfilippo C fibroblasts, although no enzyme activity recovery was observed. Using the *in vitro* TNT system the best result was obtained for the p.W168X mutation of the *SMPD1* gene treated with G418, reaching a 35% recovery of the full-length protein. Finally, gentamicin treatment of COS cells transfected with mutant *ARSB* cDNA carrying the p.W146X mutation showed a recovery of enzyme activity of around two-fold.

In general, our results and those of others on readthrough treatment for nonsense mutations show a certain degree of recovery either in protein levels, mRNA levels or enzyme activity. The results are sometimes inconsistent between groups, mutations, compounds and techniques. However, the positive results are promising and, in some cases, they have led to clinical trials, the results of which will have important implications for the field. The small molecule readthrough (SMRT) chemicals also hold promise for systemic use, including for treatment of central nervous system involvement. Novel compounds are being generated by different groups in the search for more efficient and less toxic drugs. The fact that slight recovery of protein levels could be enough to cure these diseases, and that any good compound could be used for many diseases, is sufficient to encourage further research.
